# Supplementary material for: Silencing Dicer-Like Genes Reduces Virulence and sRNA Generation in Penicillium italicum, the Cause of Citrus Blue Mold
Source: Cells. 2020 Feb 4;9(2):363. doi: 10.3390/cells9020363 (PMC7072147; doi:10.3390/cells9020363)
Supplement: Supplementary file 1 [file cells-09-00363-s001.zip › Figure supplementary file 1 copy.pdf]

Supplementary Figure S1

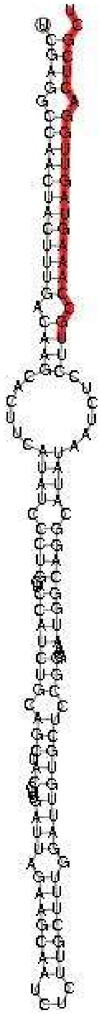

The secondary structure of precursor of Pi-novel1

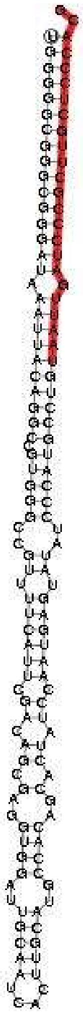

The secondary structure of precursor of Pi-novel2

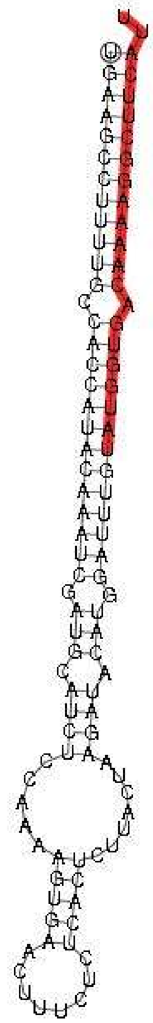

The secondary structure of precursor of Pi-novel3

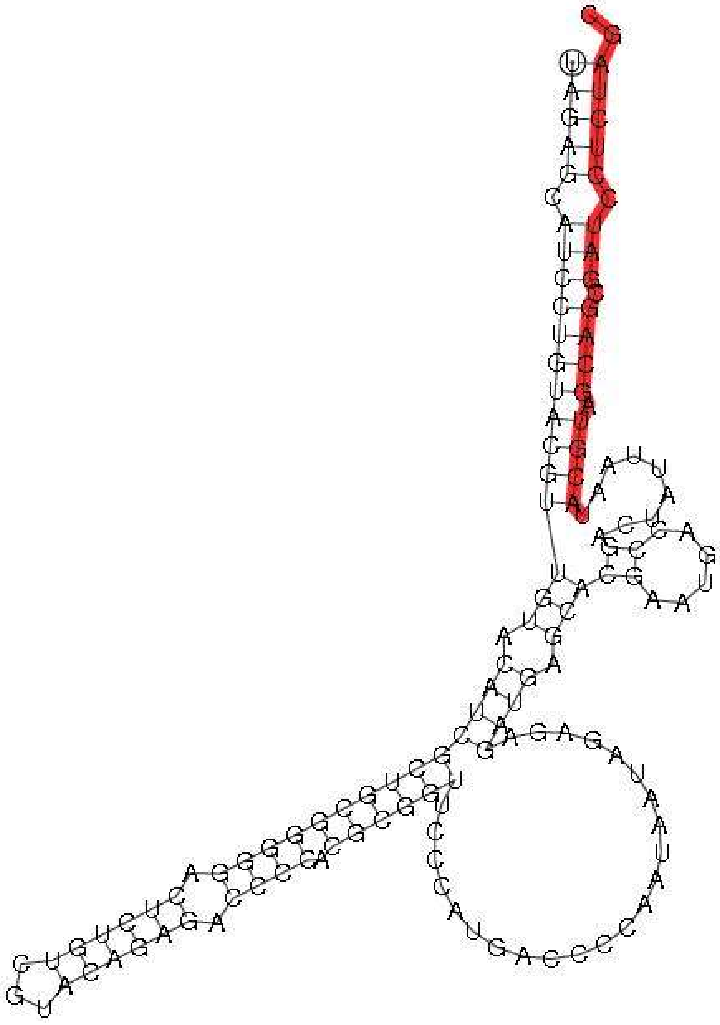

The secondary structure of precursor of Pi-novel6

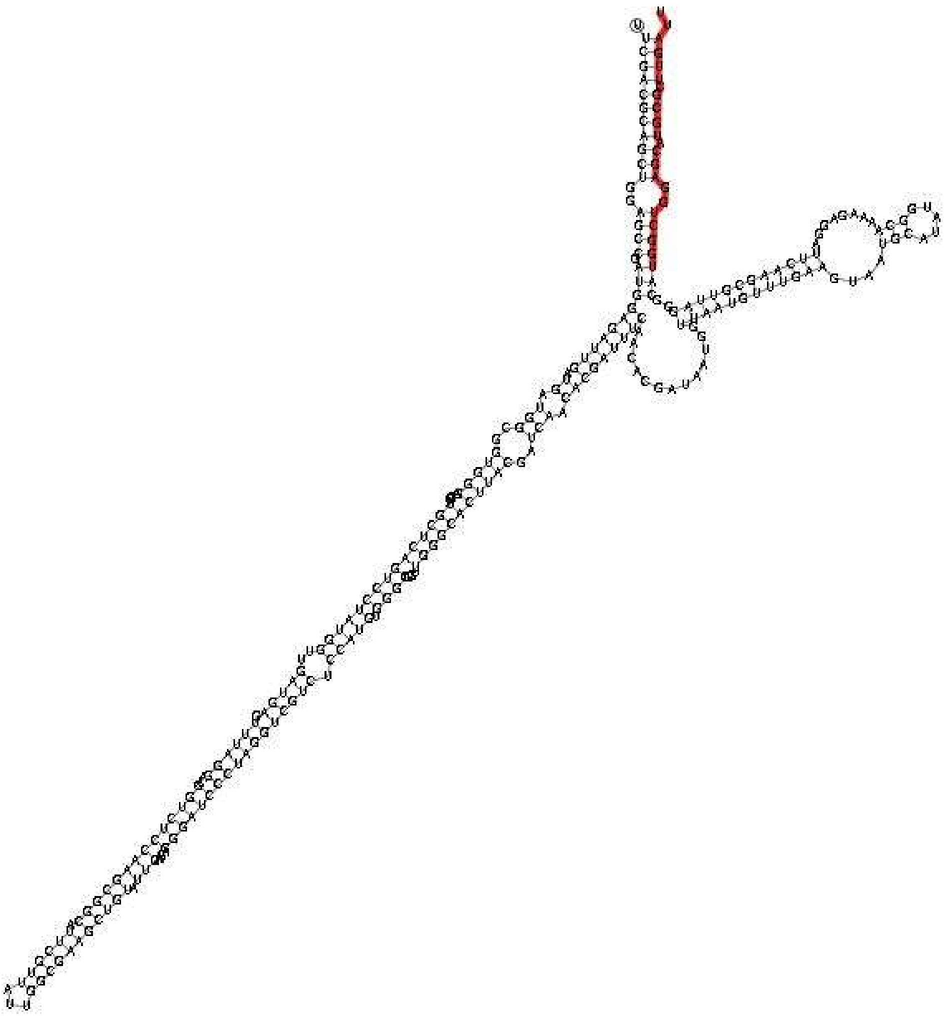

The secondary structure of precursor of Pi-novel7

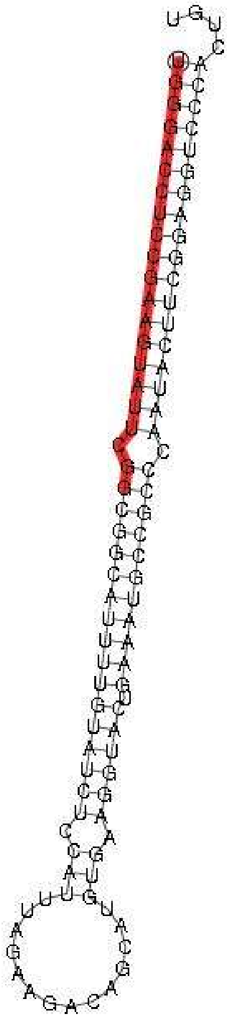

The secondary structure of precursor of Pi-novel10

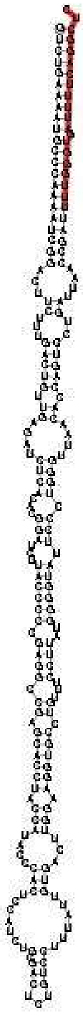

The secondary structure of precursor of Pi-novel11

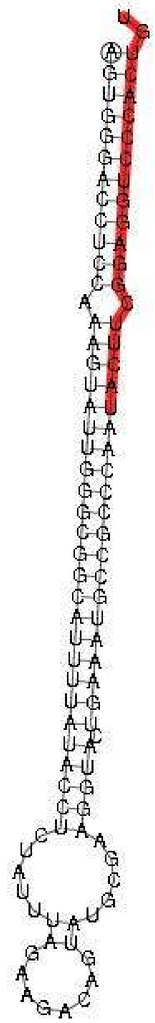

The secondary structure of precursor of Pi-novel12

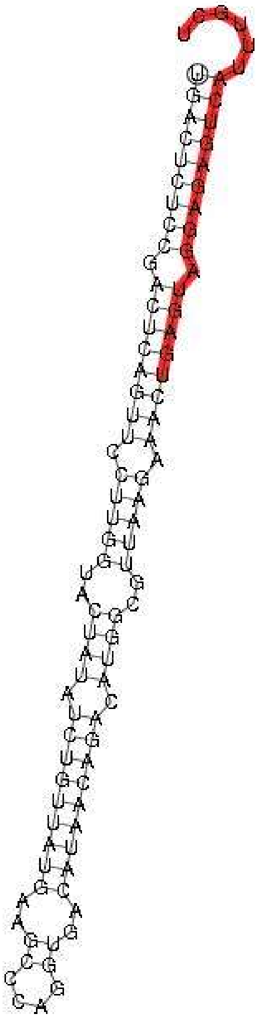

The secondary structure of precursor of Pi-novel14

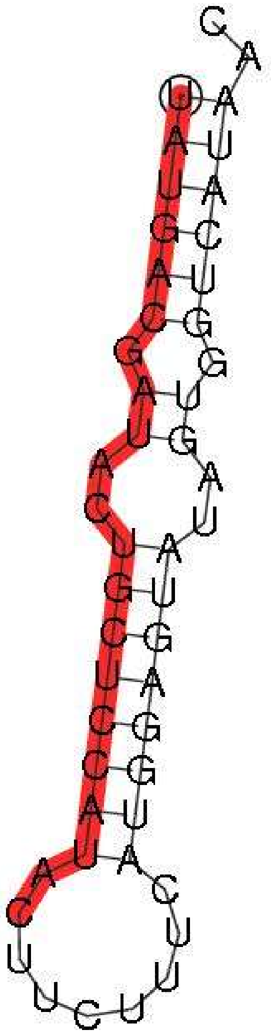

The secondary structure of precursor of Pi-novel16

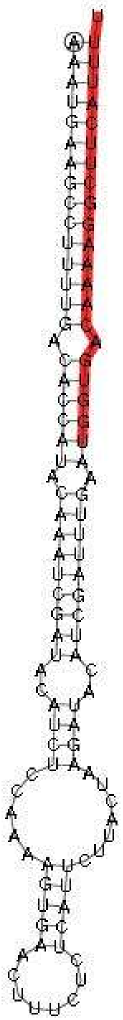

The secondary structure of precursor of Pi-novel21

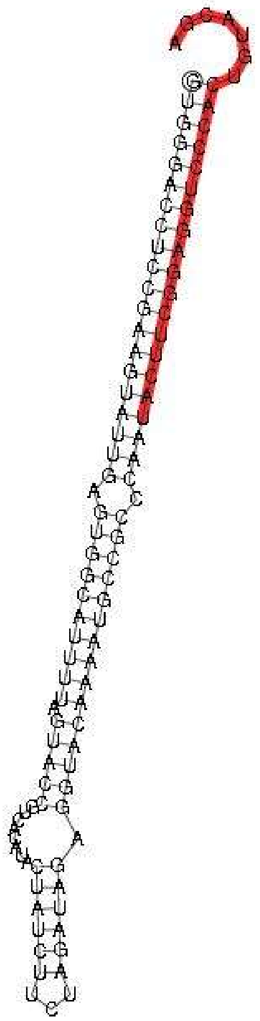

The secondary structure of precursor of Pi-novel24
