# Supplementary material for: Silencing Dicer-Like Genes Reduces Virulence and sRNA Generation in Penicillium italicum, the Cause of Citrus Blue Mold
Source: Cells. 2020 Feb 4;9(2):363. doi: 10.3390/cells9020363 (PMC7072147; doi:10.3390/cells9020363)
Supplement: Supplementary file 1 [file cells-09-00363-s001.zip › Table supplementary file 1 copy.pdf]

Table S1 Primer sequences used in this study

Primers	Sequences
QDCL1-F	5' AAAACTGCAACTGAACTGAAGCTA 3'
QDCL1-R	5' TTTCGGCTAAGGGGTGCTT 3'
QDCL2-F	5' CATGGTCGTCATGGCAGTTATCGC 3'
QDCL2-R	5' CGTGGGAAGGCTCATCTGGTGC 3'
hph-F	5' CGGAGACGCTGTCGAACTTT 3'
hph-R	5' GCTATGACCATGATTACGCCAA 3'
plus strand DCL1-F (XhoI)	5' GATACCGTCGACCTCGAGCCTCAACCGTTGGTTATA 3'
plus strand DCL1-R (HindIII)	5' GTATCCTCCAGCAAGCTTCCATACTCAAGCGAAAGA 3'
minus strand DCL1-F (KpnI)	5' GCTAAGGCCTGTGGTACCCCATACTCAAGCGAAAGA 3'
minus strand DCL1-R (ApaI)	5' TAAGTGGATCCGGGGCCCCCTCAACCGTTGGTTATA 3'
plus strand DCL2- F (XhoI)	5' GATACCGTCGACCTCGAGCTTGGCAGATGTTGTGGA 3'
plus strand DCL2- R (HindIII)	5' GTATCCTCCAGCAAGCTTCAGGAGGTTAGCATTGACG 3'
minus strand DCL2-F (KpnI)	5' GCTAAGGCCTGTGGTACCCAGGAGGTTAGCATTGACG 3'
minus strand DCL2- R (ApaI)	5' TAAGTGGATCCGGGGCCCCTTGGCAGATGTTGTGGA 3'
Pit-DCL2-F	5' TAATACGACTCACTATAGGGCTTGGCAGATGTTGTGGA 3'
Pit-DCL2-R	5' TAATACGACTCACTATAGGGCAGGAGGTTAGCATTGACG 3'
Pit-GFP-F	5' TAATACGACTCACTATAGGGGCGACGTAAACGGCCACA 3'
Pit-GFP-R	5' TAATACGACTCACTATAGGGCGAACTCCAGCAGGACCATG 3'
novel2-F	5' AATCAAGCGCATGCTCCAGCCATGC 3'

novel2-R	5' TTCGACGCAGCTGGAGCCGATGGAG 3'
novel6-F	5' GCTAGAGGATCGCTGCTACGTATTA 3'
novel6-R	5' TAGAGCATCCTGTACGTTGTACATC 3'
novel7-F<br>novel7-R	5' AATCAAGCGCATGCTCCAGCCATGC 3'<br>5' TTCGACGCAGCTGGAGCCGATGGAG 3'
novel11-F	5' GAGCCTGAAAATCACCCAAAA 3'
novel11-R	5' GTCTGAAAATGCCCAAAATCGGACT 3'
novel14-F	5' AGCAAATGACTCTCCTACTCAGTTT 3'
novel14-R	5' TGACTCTCCGACTCAGTTCCTTGGT 3'
U6	5' TGTCATCCTTGCGCAGGGGCCATGCTAATCTTCTCTGTAT 3'
novel7	5' AATCAAGCGCATGCTCCAGCCA 3'
Actin-F	5' GCACGGAAGACCTGGATT 3'
Actin-R	5' GGTTCGGTGAGTAGGAAGTAG 3'

Note: The revised primers (QDCL1-R and QDCL2-R) were designed out of the dsDCL region to avoid the amplification of the exogenous dsDCL constructs in the qRT-PCR analysis.
